# CD8 infiltration is associated with disease control and tobacco exposure in intermediate-risk oropharyngeal cancer

**DOI:** 10.1038/s41598-019-57111-5

**Published:** 2020-01-14

**Authors:** J. O. Kemnade, H. Elhalawani, P. Castro, J. Yu, S. Lai, M. Ittmann, A. S. R. Mohamed, S. Y. Lai, C. D. Fuller, A. G. Sikora, V. C. Sandulache

**Affiliations:** 1 Department of Medicine, Section of Hematology Oncology, Baylor College of Medicine, Houston, TX USA; 2 Department of Radiation Oncology, University of Texas MD Anderson Cancer Center, Houston, TX USA; 3 Department of Pathology and Immunology, Baylor College of Medicine, Houston, TX USA; 4 Bobby R. Alford Department of Otolaryngology- Head and Neck Surgery, Baylor College of Medicine, Houston, TX USA; 5 Department of Head and Neck Surgery, University of Texas MD Anderson Cancer Center, Houston, TX USA; 6 Operative Care Line, Michael E. DeBakey Veterans Affairs Medical Center, Houston, TX USA

**Keywords:** Head and neck cancer, Tumour immunology

## Abstract

Oropharyngeal squamous cell carcinoma (OPSCC) incidence is increasing at a nearly epidemic rate, largely driven by the human papillomavirus (HPV). Despite the generally favorable clinical outcomes of patients with HPV driven (HPV+) OPSCC, a significant subset of HPV tumors associated with tobacco exposure have diminished treatment response and worse survival. The tumor immune microenvironment (TIME) has been shown to be a critical driver of treatment response and oncologic outcomes in OPSCC generally and HPV+ OPSCC more specifically. However, the impact of tobacco exposure on the TIME in OPSCC patients remains unclear. We analyzed the relationship between TIME, tobacco exposure and clinical outcomes in OPSCC patients (n = 143) with extensive tobacco exposure (median pack-years = 40). P16 overexpression, a surrogate marker of HPV association, was a strong predictor of relapse-free (RFS) and overall survival (OS) (p < 0.001, p < 0.001 respectively) regardless of tobacco exposure and associated strongly with differential infiltration of the tumor by both CD3 and CD8 lymphocytes measured via immunohistochemistry (p < 001, p < 0.001 respectively). CD3 and CD8 infiltration was a strong predictor of RFS and OS and associated strongly with disease stage (AJCC 8^th^ Edition Staging Manual). Tobacco exposure correlated significantly (p < 0.001) with decreased CD8 infiltration in p16+ OPSCC tumors. Our findings demonstrate that the HPV+ OPSCC clinical outcomes are strongly correlated with the TIME, which is potentially modulated by tobacco exposure. Immunomodulatory strategies targeting this disease in smokers must take into consideration the potential modifying effects of tobacco exposure on treatment effectiveness and clinical outcomes.

## Introduction

Tumor immunity has been shown to be a critical driver of tumorigenesis, treatment response and clinical outcomes in multiple solid tumor types including head and neck squamous cell carcinoma (HNSCC). Among HNSCC sites, SCC of the oropharynx, associated with the human papillomavirus (HPV+ OPSCC) is increasing at a nearly epidemic rate across the United States^1–6^. Virally mediated malignancies such as HPV+ OPSCC demonstrate increased tumor immunity, which is thought to contribute to differential treatment response^7–10^. This may also lead to improved response to immunomodulatory strategies in this HNSCC subtype, which is currently under investigation in a number of national and international clinical trials.

Although a majority of new OPSCC diagnoses are thought to be driven by HPV, a significant number of patients also demonstrate a history of significant tobacco exposure, a known carcinogenic risk factor for OPSCC and a critical modulator of overall patient health^1–6^. In these patients, clinical outcomes are worse compared to those of non-smoking OPSCC patients^1,5,6,11,12^. The precise mechanisms which underlie the differential clinical outcomes of patients with a history of tobacco exposure remain unclear and may in fact be multi-factorial given the complex effects of tobacco exposure^2,3,6,11,13–17^. However, there is reason to believe that tobacco exposure may be an important modulator of the tumor immune microenvironment (TIME), a critical driver of treatment response and survival in patients with solid tumors^13,18,19^.

Previous literature has shown that tobacco has profound and widespread immunomodulatory effects which remain somewhat unclear. Some of this evidence suggests a potentially inflammatory effect, some a potentially immunosuppressive effect^13,18–23^. The interaction between tobacco and TIME in HPV+ OSCC therefore remains an open question. CD8 lymphocytes are a critical component of TIME and a critical mediator of checkpoint inhibition^8–10^. Previous studies have shown that CD8 infiltration is high in HPV+ OPSCC^8–10^. In the current study we evaluated the relationship between aspects of the TIME (CD3 and CD8 lymphocytes) and clinical outcomes in intermediate risk OPSCC patients (HPV+ smokers).

## Methods

### Clinical data collection

Following approval from Baylor College of Medicine and the Michael E. Debakey Veteran’s Administration (MEDVAMC) Institutional Review Boards, we reviewed the records of Veterans with previous untreated oropharyngeal squamous cell carcinoma (SCC) between January 1, 2000 and January 1, 2012. Oropharyngeal sites included: tonsil, base of tongue, pharyngeal wall, glossopharyngeal sulcus and soft palate. Waiver of consent was obtained from the IRBs; informed consent cannot be obtained from patients who are deceased and thus our normal IRB protocol is to provide a waiver of consent for retrospective analysis of previously collected tissue specimens. Data collection and analysis were performed in a manner consistent with existing standards for clinical research (Declaration of Helsinki, US Federal Policy for the Protection of Human Subjects). Inclusion criteria included primary oropharyngeal SCC, tissue diagnosis at the MEDVAMC, sufficient pathologic tissue for immunohistochemical analysis, and treatment delivery at the MEDVAMC. Exclusion criteria included treatment at an outside institution and recurrent disease at presentation. Demographic information was recorded including age, gender, race, smoking history and alcohol consumption. Smoking history is collected at our institution at the time of initial diagnosis as “pack-years” consistent with general practice. Clinical-pathologic features were collected including clinical stage according to the American Joint Commission on Cancer (Staging Manual 7^th^ Edition) staging system; staging was also recalculated using the 8^th^ Edition of the Staging Manual in order to more appropriately account for the impact of p16/HPV status and reflect current staging algorithms^24^. Results of diagnostic procedures including imaging results, biopsies, and fine needle aspirations as well as the treatments provided and the associated dates were recorded.

### Immunohistochemical analysis

Formalin-fixed, paraffin-embedded (FFPE) biopsy tissue blocks were retrieved from the archive maintained at the MEDVAMC Department of Pathology under BCM IRB and MEDVAMC R&D approved protocols. Sections were cut from each FFPE block at 5 µm thickness and mounted on positively charged slides. One section was stained with Hematoxylin & Eosin and reviewed by a surgical pathologist to confirm the original histopathological diagnosis and to ensure tumor adequacy. Slides were deparaffinized in Bond Dewax Solution (Leica Biosystems, Buffalo Grove, IL), and rehydrated in descending grades of 100%, 90% and 70% ethanol. Endogenous peroxide activity was blocked by pretreatment with 3% hydrogen peroxide for 10 minutes, and antigen recovery was achieved by 20 minutes of heat-induced epitope retrieval. Immunohistochemical (IHC) stains were performed on an automated tissue-staining system using the Bond Polymer Refine Detection (Leica Biosystems, Buffalo Grove, IL). Serial tissue sections were stained with monoclonal antibodies: CD3, clone LN10; CD8, clone 4B11; CD68, clone 514H12 (Leica, Biosystems, Buffalo Grove, IL,); p16, clone E6H4 (Ventana, Tucson, Arizona). Detection was done using polymer reagent conjugated with horseradish peroxidase followed by the 3,3′-Diaminobenzidine (DAB) Chromogen Kit (Leica Biosystems, Buffalo Grove, IL). Appropriate positive and negative controls were performed.

Low-power images were capture with 4X/0.16 objective using Vectra 3 multispectral camera. High powered images were selected using PhenoChart software (Perkin Elmer, Waltham, MA). Up to 10 fields of view were selected from each low-power image and were captured using 20X/0.75 objective. Germinal centers were excluded from high-power imaging. inForm software (Perkin Elmer, Waltham, MA) was used to analyze the high-power images. The software was trained on representative images for tissue and cell segmentation. Lymphocytes were scored using the Phenotyping feature to determine positively stained cells and cell counts were performed. P16 staining was performed as previously described by our group and classified as either positive or negative. A subgroup was defined by high-nuclear p16 staining consistent with our previous analysis^25^. Data for CD3 and CD8 were calculated as a % of positively stained nuclei compared to total nuclei.

### Study endpoints and statistical analysis

Endpoints of interest included time to recurrence and death, all reported in months. Imaging was used as a surrogate in the absence of a pathological report documenting recurrence whether local, regional or distant. ‘Relapse-free survival (RFS)’ was defined as time from date of primary diagnosis to date of diagnosis of loco-regional and/or distant recurrence (i.e. whichever was diagnosed first); ‘Overall survival (OS)’ was defined  as time from date of primary diagnosis to date of death or last recorded follow-up in hospital notes. Patients suspected of recurrence were restaged using clinical exam and imaging. Patients who did not receive curative intent (n = 16) were removed from the RFS analysis; all patients were included in the OS analysis. Actuarial survival rates were generated using the Kaplan-Meier method, and comparisons between groups were made using log-rank statistics. Chi-square test was used to report likelihood of association between categorical variables. Univariate analysis was performed using Cox regression. All variables with p ≤ 0.05 on univariate analysis were used for multivariate analysis. We applied recursive partitioning analysis (RPA) to quantify a threshold for CD3 and CD8 infiltration -as surrogates for TIME- that is significantly associated with overall survival (p < 0.05). For multivariable analysis, RFS and OS modeling using standard clinical variables (i.e. age, sex, AJCC 7^th^ & 8th edition, p16 status ascertained via immunohistochemistry, treatment modalities, and tobacco exposure) was done. For all statistics, p-values were considered to be statistically significant if below a threshold of 0.05 (two-sided). Statistical calculations were performed with JMP Pro statistical software (version 14.0; SAS Institute Inc, Cary, NC).

## Results

### Patient, tumor and treatment characteristics

We previously published clinical outcomes for a larger patient cohort treated at our institution during this time period^5^. The patients included in the current analysis represent a subset of patients for which tissue was available for detailed pathologic analysis. Therefore, we re-present the clinical data for context and because the cohort is smaller than the previously reported dataset. The full patient cohort (n = 143) consisted of 83 p16-positive (p16+) and 60 p16-negative (p16−), newly diagnosed, oropharyngeal carcinomas. All patients were male and the mean age at presentation was comparable between the two subgroups. Mean and median follow-up times for the p16+ cohort were 41.5 and 30.8 months respectively. Mean and median follow-up times for the p16− cohort were 14.9 and 7.8 months respectively. A majority of patients in both subgroups were treated with curative intent external beam radiation therapy (EBRT) based treatment though the proportion of patients receiving such treatment was higher in the p16+ compared to the p16− cohort (95% vs. 80% for curative intent, 90% vs. 67% for radiation therapy, respectively). The full cohort had a high prevalence of tobacco exposure (91%) with all p16-negative patients having used tobacco products in their lifetime (Table 1). Additionally, for most patients, this exposure was more than 30 pack-years (Supplementary Fig. 1). Alcohol consumption data was available for 141 patients. Of these 89% reported a history of alcohol exposure prior to diagnosis. More detailed quantification of timing of exposure and exposure amount was not available for this patient cohort.

**Table 1:** Patient, tumor and treatment characteristics.

		p16+ (n = 83)	p16− (n = 60)
Age (years)	mean	59.8	60.8
Gender	male	83	60
	female	0	0
Race	black	16	16
	white	66	44
	other	1	0
Tobacco use	yes	70	60
	no	13	0
T-classification	1	17	4
	2	27	18
	3	17	21
	4	22	17
N-classification	0	14	25
	1	7	6
	2a	6	3
	2b	34	9
	2c	18	12
	3	4	5
M-classification	0	80	56
	1	3	4
Treatment	curative	79	48
	non-curative	4	12
	primary EBRT	75	40
	adjuvant EBRT	2	4
Vital Status at 5 years	Alive	53	16
	Deceased	30	44

### Impact of AJCC status, p16, and tobacco exposure on survival

**Figure 1:**
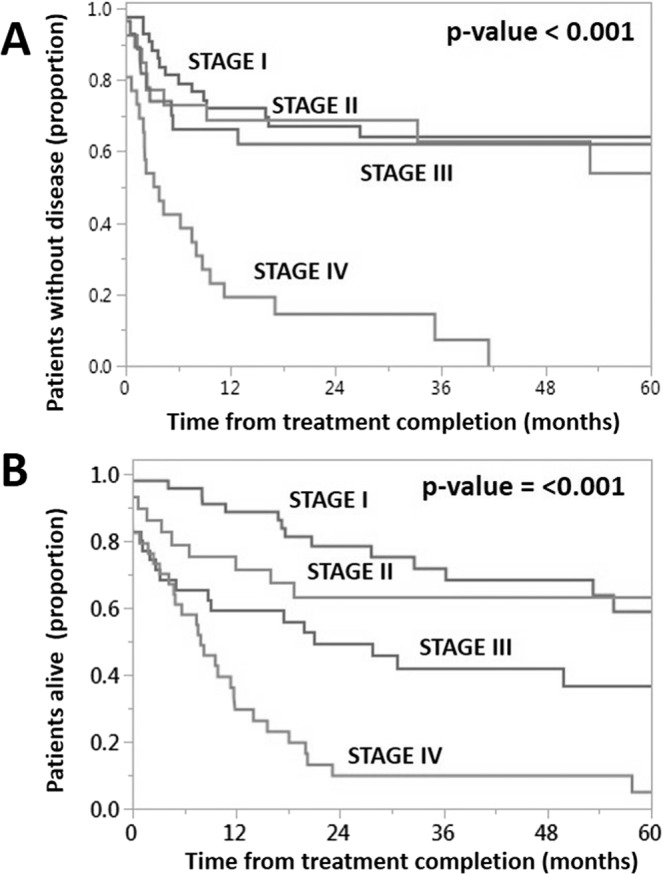
Impact of AJCC staging on survival. Relapse free survival (**A**) and overall survival (**B**) stratified by AJCC 8^th^ Edition Staging Manual. Kaplan-Meier curves, p-value generated using log-rank analysis.

**Figure 2:**
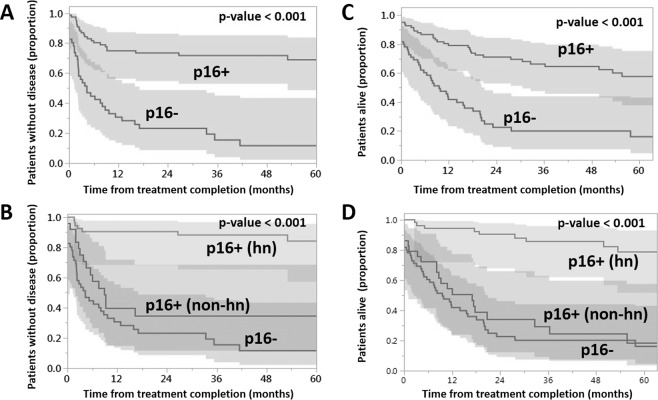
Impact of p16 on survival. Relapse free survival as a function of p16 status (negative vs positive (**A**); negative vs positive (high-nuclear (hn) staining) vs positive (non high-nuclear (hn) staining (**B**). Overall survival as a function of p16 status (negative vs positive (**C**); negative vs positive (high-nuclear (hn) staining) vs positive (non high-nuclear (hn) staining (**D**). Kaplan-Meier curves, p-value generated using log-rank analysis.

Several non-immune related tumor characteristics correlated well with both RFS and OS. In univariable analysis, N-classification (ICON-S), AJCC (8^th^ edition), and P16 status were significantly associated with RFS while T-classification, N-classification, M-classification, AJCC stage (8^th^ edition) p16, and tobacco exposure (in pack-years), were significantly correlated with OS (Tables 2 and 3). AJCC 7^th^ Edition Staging Manual classification demonstrated no correlation between disease stage and either RFS or OS (Supplementary Fig. 2) while re-classification to tumor stage using the AJCC 8^th^ Edition Staging Manual improved separation across stages for both RFS and OS compared to the AJCC 7^th^ Edition Staging Manual (Fig. 1). P16 status impacted both RFS and OS (Fig. 2). Additionally, using a previously described scoring system^25^, high-nuclear p16 staining tumors were associated with a significantly improved RFS and OS (Fig. 2). The preferentially higher survival rates for patients with high-nuclear p16 staining was maintained even on multivariate analysis (Tables 2 and 3), with non high-nuclear and negative p16 tumors demonstrating similar hazard ratios for RFS and OS.

**Table 2:** Univariate and multivariate analysis for predictors of relapse-free survival.

Variable	Variable categories	Univariate Analysis		Multivariate Analysis	
		Hazard ratio (95% CI)	p-value	Hazard ratio (95% CI)	p-value
p16	HN (high nuclear)	—		—	
	non-HN	7.39 (3.09–19.51)	<0.0001	8.25 (3.36–22.36)	<0.0001
	negative	12.39 (5.79–30.72)	<0.0001	8.73 (3–27.84)	<0.0001
Tobacco (pack-years)			0.2		
CD3 infiltrate	≥32.89%	—		—	
	<32.89%	3.6 (1.9–7.5)	<0.0001	2.1 (1.05–4.7)	0.03
CD8 infiltrate	≥7%	—		—	
	<7%	2.2 (1.3–3.6)	0.006	1.1 (0.6–1.9)	0.8
Age (continuous)			0.35		
Race	White	—			
	Others	0.99 (0.5–1.7)	0.96		
T - classification	T1–2	—			
	T3–4	1.2 (0.7–2)	0.41		
N - classification	N0–1	—			
	N2–3	1.3 (0.8–2.2)	0.27		
AJCC 7^th^ Edition			0.16		
AJCC 8^th^ Edition	I	—		—	
	II	1.2 (0.5–2.7)	0.6	1.01 (0.4–2.5)	0.9
	III	1.2 (0.5–2.7)	0.6	0.7 (0.3–1.6)	0.4
	IV	5.1 (2.7–9.9)	<0.0001	1.5 (0.6–4.1)	0.4

Note: p16 status = p16 status assessed via immunohistochemistry.

**Table 3:** Univariate and multivariate analysis for predictors of overall survival.

Variable	Variable categories	Univariate Analysis		Multivariate Analysis	
		Hazard ratio (95% CI)	p-value	Hazard ratio (95% CI)	p-value
P16 status	HN (high nuclear)	—		—	
	non-HN	7.06 (3.26–16.48)	<0.0001	7.86 (3.44–19.16)	<0.0001
	negative	8.59 (4.34–18.99)	<0.0001	6.91 (3.19–16.48)	<0.0001
Tobacco (pack-years)			0.046		0.5
CD3 infiltrate	≥32.89%	—		—	
	<32.89%	3.1 (1.7–6.1)	<0.0001	1.3 (0.7–2.9)	0.4
CD8 infiltrate	≥7%	—			
	<7%	3.02 (1.9–4.8)	<0.0001	1.9 (1.1–3.3)	0.01
Age			0.35		
Race	White	—			
	Others	0.96 (0.5–1.6)	0.9		
T - classification	T1–2	—			
	T3–4	1.9 (1.2–3)	0.007		
N - classification	N0–1	—			
	N1–2	1.6 (1.01–2.6)	0.045		
M - classification	M0				
	M1	3.81 (1.32–8.72)	0.02		
AJCC 7^th^ Edition			0.55		
AJCC 8^th^ Edition	I	—		—	
	II	1.43 (0.61–3.33)	0.4	2.9 (1.1–7.6)	0.02
	III	2.66 (1.31–5.6)	0.006	3.2 (1.4–7.5)	0.005
	IV	6.02 (3.1–12.42)	<0.0001	5.9 (2.2–16.8)	0.0003

Note: p16 status = p16 status assessed via immunohistochemistry.

### Impact of CD3 and CD8 infiltration on survival

Stratification of tumors by immune infiltrate as a function of total nuclear count demonstrated a significant disease-free and overall survival advantage for patients with more infiltrated tumors (Supplementary Fig. 3). Using Kaplan-Meier analysis coupled to log-rank test, we identified statistically significant correlations between CD3 and CD8 infiltration with both RFS and OS. Based on these results, we then performed a recursive partitioning analysis to identify CD3 and CD8 infiltration thresholds which impact RFS and OS.

**Figure 3:**
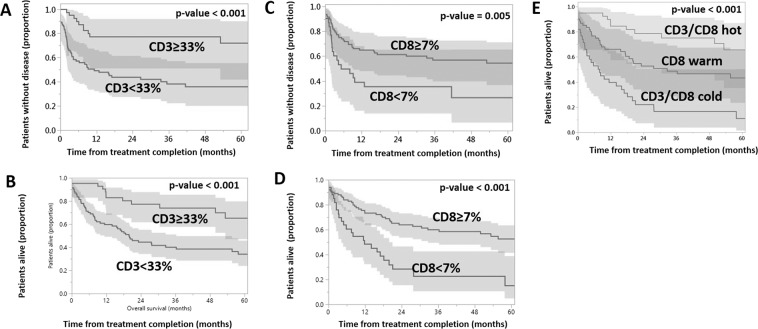
Impact of CD3 and CD8 infiltration on survival. Relapse free survival as a function of CD3 (**A**) and CD8 (**C**) infiltration. Overall survival as a function of CD3 (**B**) CD8 (**D**) and CD3/CD8 infiltration (**E**). Kaplan-Meier curves, p-value generated using log-rank analysis.

Recursive partitioning analysis (RPA) assessing RFS and OS as a function of mean CD3 and CD8 infiltration treated as continuous variables, detected a statistically significant effect on RFS and OS at a CD3 threshold of 33% and a CD8 threshold of 7% (Fig. 3). Combining the CD8 and CD3 infiltrate score into one classifier (termed CD3/CD8 classifier) allowed for the creation of three sub-categories of “cold” (low CD3 and CD8 infiltrate), “warm” (high CD8 only infiltrate), and “hot” (high CD3 and CD8 infiltrate) tumors with correspondingly increasing OS (Fig. 3E). Based on these data, CD3 and CD8 infiltration (using the RPA generated thresholds) was incorporated into univariable and multivariate analysis on the dataset (Tables 2 and 3). Univariable analysis demonstrated that both CD3 and CD8 infiltrates were significantly associated with RFS (p < 0.0001 and 0.006, respectively) and OS (p < 0.0001 for both). On multivariate analysis CD3 infiltration was associated with RFS (p = 0.03) and CD8 infiltration was associated with OS (p = 0.01).

Overall, p16+ tumors demonstrated a near two-fold increase in both CD3 and CD8 infiltration (Table 4). CD3/CD8 infiltration of tumors using the combined classifier was also strongly correlated (likelihood ratio; p < 0.001) with p16 status. Lymphocyte-rich (“hot”) tumors are overrepresented in the p16+ cohort while lymphocyte-poor (“cold”) tumors are predominantly found in the p16− cohort (Supplementary Fig. 4A). When analysis was limited to patients with high-nuclear p16+ staining tumors treated with curative intent, CD8+ infiltration remained correlated to RFS (Supplementary Fig. 5). CD3/8 combined classifier also correlated strongly (likelihood ratio; p < 0.001) with the AJCC Staging Manual 8^th^ Edition staging (Supplementary Fig. 4B).

**Table 4:** p16 impact on lymphocyte infiltration.

CD3+ infiltrate (%)	All patients	p16+	p16−	p16+ vs p16− (t-test)
mean	24.7	30.6	16.6	*P* < *0.001*
median	21.5	28.0	12.5	*P* < *0.001*
**CD8+ infiltrate (%)**	**All patients**	**p16+**	**p16−**	**p16+ vs p16− (t-test)**
mean	13.3	16.6	8.7	*P* < *0.001*
median	9.9	14.0	7.2	*P* < *0.001*

### Interaction between tobacco exposure and TIME

**Figure 4:**
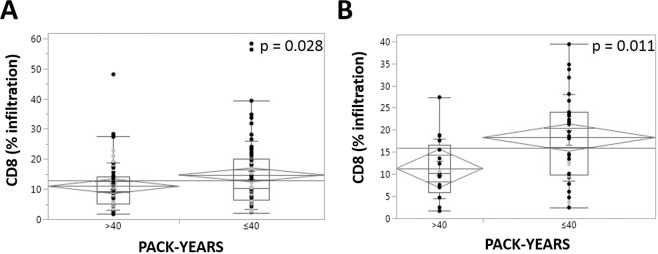
Impact of tobacco exposure on CD8+ infiltration. Relative CD8 infiltration as a function of tobacco exposure (median = 40 pack years) for the entire patient cohort (**A**) and for the cohort of patients with high-nuclear p16 staining (**B**).

Stratification of patients by tobacco exposure history suggested a possible link between increased tobacco exposure and decreased tumor immune infiltrate. Specifically, in the high-nuclear p16+ staining tumors, increased tobacco exposure was associated with a statistically significant decrease in CD8+ infiltration (Table 5). When we re-analyzed the same data-set using median pack-year exposure as a threshold, we detected the same decrease in CD8 infiltration (Fig. 4). This effect of tobacco exposure was maximal in the high-nuclear p16 staining tumors and was no longer significant for p16- or non high-nuclear staining tumors.

**Table 5:** Impact of tobacco exposure on lymphocyte infiltration in p16 positive tumors.

CD3+ infiltrate (%)	<30 pack-years	>30 pack-years	p-value (t-test)
mean	33.3	28.4	0.288
median	30.4	31.0	0.388
minimum	6.8	3.7	0.140
maximum	65.7	63.5	0.651
**CD8+ infiltrate (%)**	<**30 pack-years**	**>30 pack-years**	**p-value (t-test)**
mean	19.0	13.4	***0.035***
median	19.6	11.4	***0.022***
minimum	3.6	1.7	0.205
maximum	39.4	34.8	***0.052***

## Discussion

Modern day oncologic outcomes for HPV+ OPSCC are remarkable, considering the relative lack of clinical progress in other HNSCC disease sites^26,27^. Unlike other sites in which survival has remained relatively flat over the last 2–3 decades (oral cavity) or even decreased (larynx), survival for patients with OPSCC continues to improve, driven primarily by a shift in the epidemiology of the disease toward HPV mediated cancers^2,26–29^. However, this shift in survival outcomes for patients with OPSCC has been largely uneven across different segments of the US population^12,30^. One prime culprit for this differential response is tobacco exposure. Multiple datasets have shown that HPV status and tobacco exposure interact strongly to modulate treatment response and patient survival^1^. A recent analysis demonstrated a deleterious effect of tobacco exposure on HPV+ OPSCC outcomes of more than 20%, a dramatic effect that compares favorably to several known prognostic indicators such as positive cervical nodes, extranodal extension and the addition of chemotherapy to adjuvant radiation^6,27,31,32^. This is consistent with post-hoc analyses of several prospective clinical trials including RTOG0129 and RTOG9003^15^. A parallel analysis by our group has identified a similar pattern of inferior oncologic outcomes in HPV+ OPSCC patients with extensive tobacco exposure (≥30 pack-years) (manuscript submitted). We previously showed that in Veterans with heavy tobacco exposure (a majority)^5,33^, survival for HPV+ and HPV− disease were in line with the rates reported by Ang *et al*. for intermediate-risk and high-risk disease^5,34^. This effect was consistent across racial groups^35^, and matches long term results from both RTOG0129 and RTOG0522^11^.

The introduction of immunomodulatory agents for OPSCC represents both an opportunity to improve clinical outcomes and a challenge in terms of stratifying patients to the appropriate treatment regimen and intensity^36–40^. Several clinical trials, including CheckMate 141 have now demonstrated substantial effectiveness of immunomodulatory agents both in the single agent setting and when combined with chemotherapy with a potentially more pronounced effect in p16+ tumors^36,38^. More recently pembrolizumab was shown to increase survival in the first line setting for recurrent or metastatic HNSCC either alone in the PD-L1 combined positive score (CPS) positive population or combined with chemotherapy in the CPS negative population when compared to cetuximab and chemotherapy combination therapy^41^. It is very likely that TIME will be a critical driver of differential efficacy for immunomodulatory strategies. Therefore, modifiers of TIME are likely to impact relative efficacy and will need to be considered during regimen design and trial implementation.

Tobacco exposure has potent effects on anti-tumor and systemic immunity and inflammation^42^, including direct modulation of immunocyte gene expression and function^19,21,23^. These effects are broad based and are not completely consistent across solid tumor types as shown in a recent TCGA analysis of head and neck and lung tumors^13^. A recent analysis of several datasets from oral cavity SCC (OCSCC) demonstrated that changes in the tumor immune microenvironment were the primary effect noted secondary to variable tobacco exposure^43^. The current analysis extends these previous datasets in two overlapping ways. First, we demonstrated that in OPSCC patients with extensive tobacco exposure, RFS strongly correlates with CD3 tumor infiltration on both univariable and multivariate analysis and with CD8 on univariable analysis. Additionally, OS strongly correlates with CD8 tumor infiltration on both univariable and multivariate analysis and with CD3 on univariable analysis. These correlations hold in the context of p16 positivity, especially for patients who demonstrate high-nuclear p16 staining, suggesting that in patients in which p16 staining correlates with putative functional implications (regulation of cellular processes) TIME may be a critical driver of overall tumor biology and treatment response. Overall, the correlation between TIME and survival in a cohort enriched for intermediate-risk patients (smokers) is encouraging, since it indicates that potentially immunomodulatory strategies targeting tumor lymphocyte infiltration and activation might achieve a favorable effect even in patients with extensive tobacco exposure. This has significant implications for planned and ongoing clinical trials which include both low-risk and intermediate-risk OPSCC in the context of immune modulation. Second, we demonstrated that tobacco exposure significantly and substantially reduces CD8 infiltration in the context of HPV+ OPSCC. Patients with p16+ tumors demonstrated approximately a 2-fold increase in CD3 and CD8 infiltrates compared to patients with p16− tumors. By way of comparison, patients with extensive tobacco exposure demonstrated nearly a 40% reduction in CD8 infiltration despite maintaining p16 positivity. Therefore, the potential effect size of tobacco-based modulation of TIME is substantial. Based on data from other tumor types and preclinical models, this would be expected to impact the effectiveness of immunomodulatory strategies targeting infiltrating lymphocytes. Clinical trials which incorporate immunomodulatory strategies as part of a de-escalation approach may be inappropriate for intermediate-risk OPSCC patients and should consider very carefully criteria for inclusion of patients^11^.

The current study has several limitations. First, the analysis is limited to a small number of immunohistochemistry markers, which is less comprehensive than high-throughput molecular approaches (Nanostring, RNAsequencing). However, immunohistochemistry also has the higher translational potential which is an advantage for replication of the data and integration into clinical practice. Second, we analyzed a patient cohort with extensive tobacco exposure which may not be representative of patients with minimal tobacco exposure. Although true, this also provides us with the additional power to detect a tobacco effect which was the primary goal of this study. Finally, our analysis cannot differentiate between direct effects of tobacco exposure on TIME and indirect effects driven by changes in tobacco induced genomic, epigenetic and proteomic changes at the level of tumors cells which secondarily impact TIME.

## Conclusion

Differential lymphocyte infiltration of HPV+ OPSCC is associated with significant differences in survival and is impacted by tobacco exposure. Mechanistic studies are required to better elucidate the mechanisms by which tobacco exposure can modulate HPV+ OPSCC TIME and may impact response to immunomodulatory strategies.

## Supplementary information

Supplementary Data.
