# Supplementary material for: CD8 infiltration is associated with disease control and tobacco exposure in intermediate-risk oropharyngeal cancer
Source: Sci Rep. 2020 Jan 14;10:243. doi: 10.1038/s41598-019-57111-5 (PMC6959290; doi:10.1038/s41598-019-57111-5)

CD8 infiltration is associated with disease control and tobacco exposure in intermediate-risk oropharyngeal cancer

Kemnade JO [1], Elhalawani H [2], Castro P [3,4], Yu J [5], Lai S [4], Ittmann M [3,4], Mohamed ASR [2], Lai SY [6], Fuller CD [2], Sikora AG [5,7], Sandulache VC [5,7]

1. Department of Medicine, Section of Hematology Oncology, Baylor College of Medicine, Houston, TX

2. Department of Radiation Oncology, University of Texas MD Anderson Cancer Center, Houston, TX

3. Department of Pathology, Baylor College of Medicine, Houston, TX

4. Department of Pathology, Michael E. DeBakey VA Medical Center, Houston, TX

5. Bobby R. Alford Department of Otolaryngology- Head and Neck Surgery, Baylor College of Medicine, Houston, TX

6. Department of Head and Neck Surgery, University of Texas MD Anderson Cancer Center, Houston, TX

7. Operative Care Line, Michael E. DeBakey Veterans Affairs Medical Center, Houston, TX

Conflicts of interest: The authors have no conflicts of interest to disclose for this manuscript.

Running title: Tumor immunity is impacted by p16 status and tobacco exposure

Keywords: oropharynx, smoking, CD8, p16, tumor immunity

Word Count: 4300

Corresponding author:

Vlad C. Sandulache MD PhD

Assistant Professor

Bobby R. Alford Department of Otolaryngology – Head and Neck Surgery

Baylor College of Medicine

1977 Butler Blvd. 5th Floor, Ste E5.200

Houston, TX 77030

Office: (713) 798-7218

Fax: (713) 798-5841

Email: vlad.sandulache@bcm.edu

Supplementary Figure Legends

Supplementary Figure 1. Distribution of tobacco exposure across the entire patient cohort.

Supplementary Figure 2. Impact of AJCC staging on survival. Relapse free survival (A) and overall survival (B) stratified by AJCC 7th Edition Staging Manual. Kaplan-Meier curves, p-value generated using log-rank analysis.

Supplementary Figure 3. Impact of tumor immune infiltrate on survival. Relapse free survival as a function of CD3+ (A) and CD8+ (C) infiltration. Overall survival as a function of CD3+ (B) or CD8+ (D) infiltration. Kaplan-Meier curves, p-value generated using log-rank analysis. Inset includes representative figures utilized to quantify relative tumor infiltration by lymphocytes. Left sided panels represent bright field pictures of immunohistochemistry (IHC) for CD3 and CD8 expressing cells respectively. Right sided panels represent pseudocolor masking of cells which staining positive (green) for CD3 or CD8 and cells which do not stain positive (red). Black arrow points to a conglomerate of CD3 positive staining cells; gray arrow points to the same cell conglomerate on the pseudocolor masked panel. Top panels are representative of tumors with high immune infiltrates, bottom panels are representative of tumors with low immune infiltrates.

Supplementary Figure 4. Correlation between CD3/CD8 classifier and p16 status (A) and AJCC stage (B).

Supplementary Figure 5. Proportion of patients free of disease as a function of CD8+ infiltration. Only patients treated with curative intent and demonstrating high nuclear p16 staining were included in the analysis. Kaplan-Meier curves, p-value generated using log-rank analysis.

SUPPLEMENTARY FIGURE 1

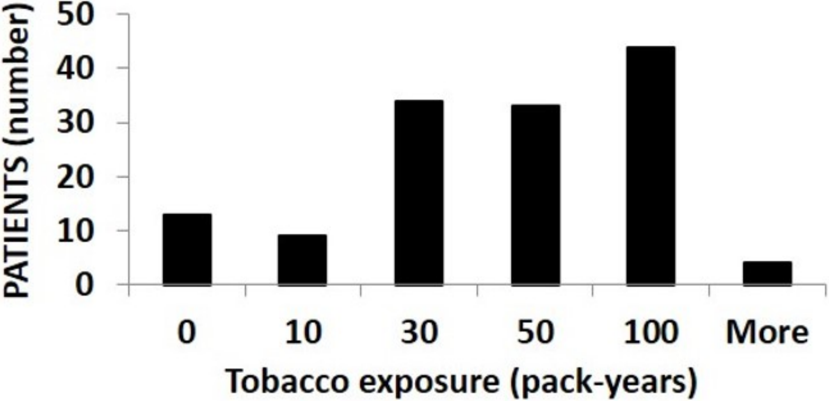

SUPPLEMENTARY FIGURE 2

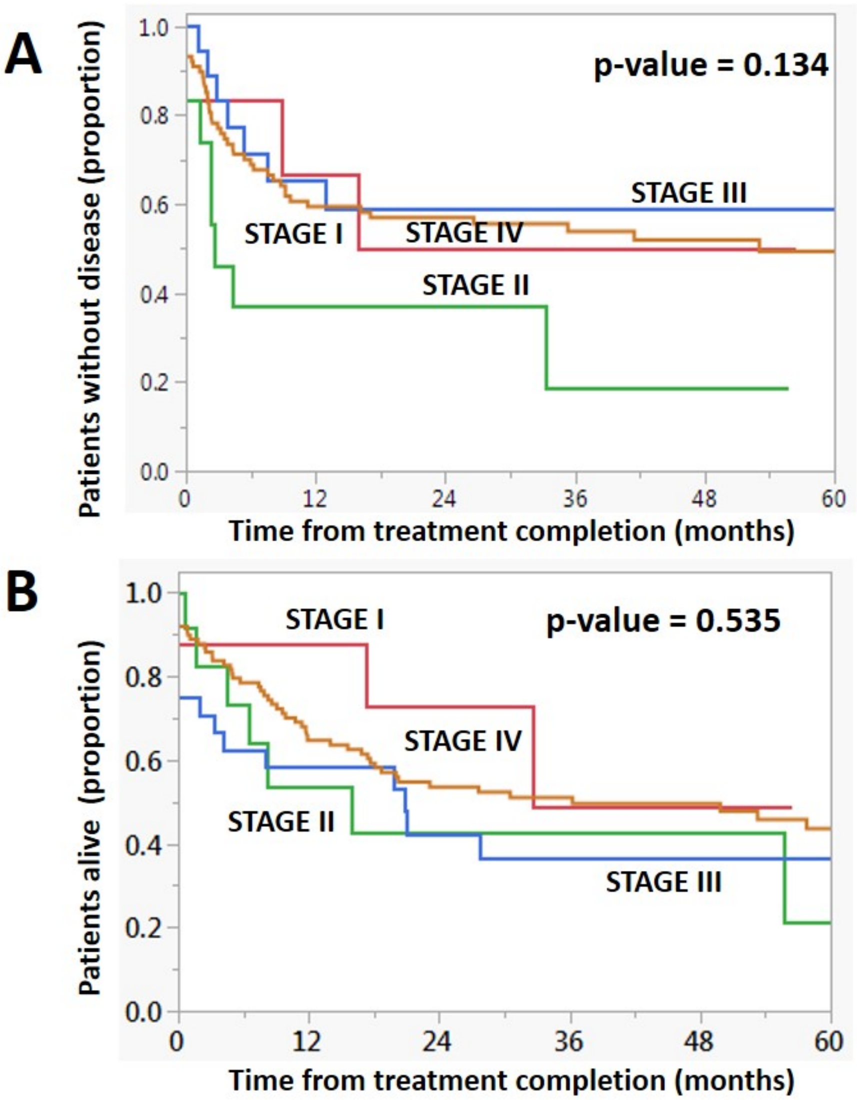

SUPPLEMENTARY FIGURE 3

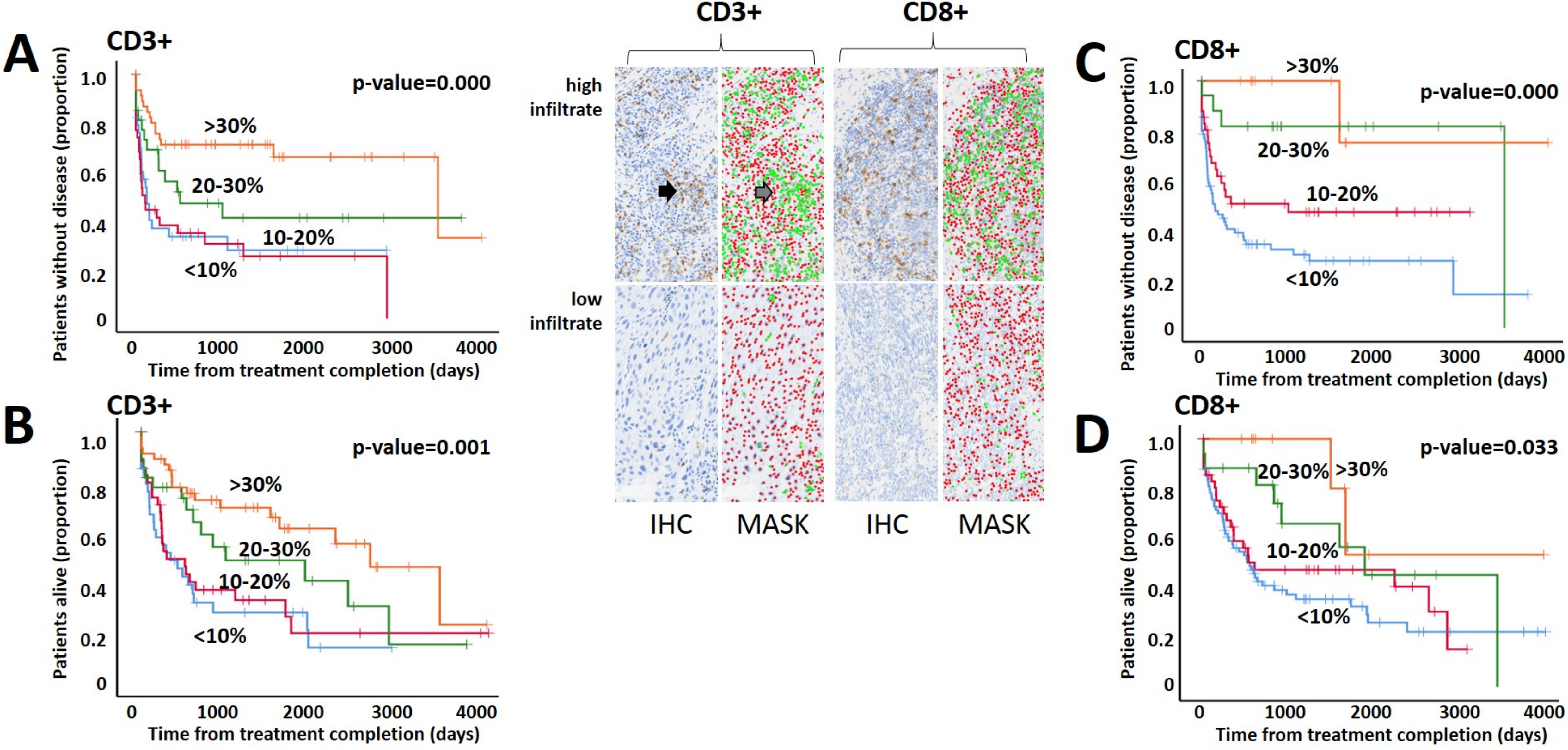

SUPPLEMENTARY FIGURE 4

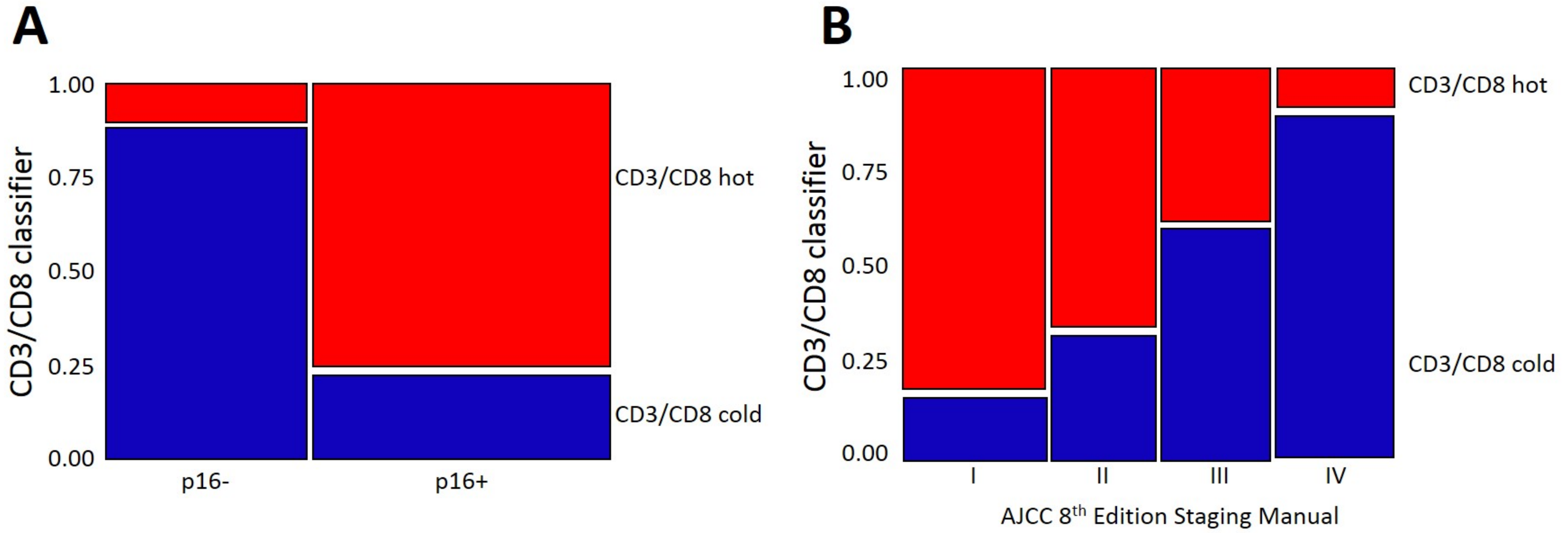

SUPPLEMENTARY FIGURE 5

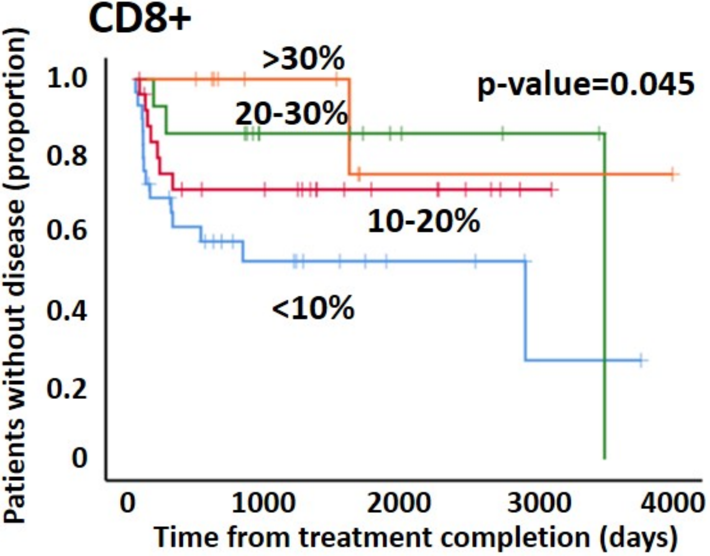

Supplement: Supplementary file 1 — Supplementary Data. [file 41598_2019_57111_MOESM1_ESM.pdf]
